# Draft Genome Sequence of *Bacillus subtilis* 2C-9B, a Strain with Biocontrol Potential against Chili Pepper Root Pathogens and Tolerance to Pb and Zn

**DOI:** 10.1128/genomeA.01502-17

**Published:** 2018-01-18

**Authors:** Claudia Y. Muñoz-Moreno, Yumiko De La Cruz-Rodríguez, Julio Vega-Arreguín, Miguel Alvarado-Rodríguez, José Manuel Gómez-Soto, Alejandro Alvarado-Gutiérrez, Saúl Fraire-Velázquez

**Affiliations:** a Biología Integrativa de Plantas y Microorganismos, Unidad Académica de Ciencias Biológicas, Universidad Autónoma de Zacatecas, Zacatecas, Mexico; b ENES-León, Universidad Nacional Autónoma de México, Mexico City, Mexico; c Unidad Académica de Agronomía, Universidad Autónoma de Zacatecas, Zacatecas, Mexico; d Unidad Académica de Matemáticas, Universidad Autónoma de Zacatecas, Zacatecas, Mexico

## Abstract

*Bacillus subtilis* 2C-9B, obtained from the rhizosphere of wild grass, exhibits inhibition against root rot causal pathogens in *Capsicum annuum*, Pb and Zn tolerance, and plant growth promotion in medium supplemented with Pb. The genome of *B. subtilis* 2C-9B was sequenced and the draft genome assembled, with a length of 4,215,855 bp and 4,723 coding genes.

## GENOME ANNOUNCEMENT

Biocontrol of phytopathogens in horticultural crops can greatly benefit from the identification of new strains of bacterial species as alternatives to counteract specific crop diseases. In addition, tolerance to heavy metals and plant growth-promoting activity are other characteristics that, if present in the isolated bacterial strain, could be of great biotechnological relevance for the bioremediation of heavy metal soil-polluted regions. *Bacillus subtilis* is well known to possess properties of phytopathogen inhibition, plant growth promotion, and heavy metal absorption (1–3); moreover, in other *Bacillus* and *Halobacillus* species, the improvement of resistance in plants to heavy metals has been reported (4, 5). In this study, we report the draft genome sequence of *Bacillus subtilis* 2C-9B, which has inhibitory activity against *Phytophthora capsici*, *Fusarium solani*, and *Rhizoctonia solani*, pathogens that cause root rot in chili pepper. Moreover, this strain shows tolerance to Pb (2,500 ppm) and Zn (400 ppm), promotes plant growth in *Arabidopsis thaliana* in medium supplemented with Pb, and synthesizes indoleacetic acid. The genome was sequenced using the MiSeq platform (Illumina, San Diego, CA, USA) in a 2 × 75 paired-end run. The genome library was prepared according to Nextera kit instructions, and the library quality was analyzed in a Bioanalyzer 2010 (Agilent Technologies). Genome assembly was performed using the SPAdes genome assembler (6), and the quality was analyzed using QUAST 4.1 (7). For genome annotation, the NCBI Prokaryotic Genome Annotation Pipeline was used (8). In the draft genome of *B. subtilis* strain 2C-9B, a total of 4,823 genes are reported, of which, 4,000 are coding genes, 100 are RNA genes (22 rRNAs, 73 tRNAs, and 5 noncoding RNAs [ncRNAs]), and 723 are pseudogenes.

Two nonribosomal peptide synthetases and a beta-glucanase gene were found in the genome of this bacterium, suggesting a role of these genes in the observed antifungal activity (9). Also, a butanediol dehydrogenase gene and a spermidine synthase gene were found, with butanediol being a potential inducer of systemic resistance in plants (10) and the spermidine gene associated with plant growth promotion (11). Although no *pbr* genes were found, other genes related to heavy metal resistance were identified by sequence homology. Among these were *zntR*, which mediates the expression of the zinc export protein *zntA*; *zntB*, which is involved in the transport of zinc (12); and a *merR* family transcriptional regulator which activates transcription in response to metal ions (13). Also, a cadmium-translocating P-type ATPase, a copper-translocating P-type ATPase, a copper-binding protein, and the copper transporter CopZ were found. Furthermore, CheA and CheC are present in the genome of this bacterium; these proteins are usually involved in chemotaxis and adaptation (14, 15). An endoglucanase and an *N*-acetylglucosamine-6-phosphate deacetylase, which are enzymes involved mainly in the consumption of carbon sources, were also found.

Considering the gene profile of this bacterial strain, *B. subtilis* 2C-9B can be seen from the perspective of a biotechnological tool with multipurpose applications, including biocontrol of phytopathogens, bioremediation of Pb- and/or Zn-contaminated areas, and increase in crop yields.

### Accession number(s).

This whole-genome shotgun project has been deposited at DDBJ/ENA/GenBank under the accession number MOXE00000000. The version described in this paper is version MOXE01000000.
